# Animal board invited review: Widespread adoption of genetic technologies is key to sustainable expansion of global aquaculture

**DOI:** 10.1016/j.animal.2022.100642

**Published:** 2022-10

**Authors:** Ross D Houston, Christina Kriaridou, Diego Robledo

**Affiliations:** a Benchmark Genetics, 1 Pioneer Building, Edinburgh Technopole, Penicuik, UK; b The Roslin Institute and Royal (Dick) School of Veterinary Studies, University of Edinburgh, Easter Bush, UK

**Keywords:** Biotechnology, Food security, Genetic technologies, Genomics, Selective breeding

## Abstract

Aquaculture production comprises a diverse range of species, geographies, and farming systems. The application of genetics and breeding technologies towards improved production is highly variable, ranging from the use of wild-sourced seed through to advanced family breeding programmes augmented by genomic techniques. This technical variation exists across some of the most highly produced species globally, with several of the top ten global species by volume generally lacking well-managed breeding programmes. Given the well-documented incremental and cumulative benefits of genetic improvement on production, this is a major missed opportunity. This short review focusses on (i) the status of application of selective breeding in the world’s most produced aquaculture species, (ii) the range of genetic technologies available and the opportunities they present, and (iii) a future outlook towards realising the potential contribution of genetic technologies to aquaculture sustainability and global food security.

## Highlights

• The extent of application of genetic technologies to aquaculture production varies widely by species and geography.
• Achieving a more universal application of seed derived from scientifically based breeding programmes is an important goal in order to meet increasing global demands for seafood production.
• This article reviews the status of genetic technologies across the world’s top 10 highly produced species.
• Opportunities and barriers to achieving broad-scale uptake of genetic technologies in global aquaculture are discussed.
• A future outlook for potential disruptive genetic technologies and how they might affect global aquaculture production is given.

## Implications

The extent of application of genetic technologies to aquaculture production varies widely by species and geography. Achieving a more universal application of seed derived from scientifically based breeding programmes is an important goal in order to meet increasing global demands for seafood production. This article reviews the status of genetic technologies across the world’s top 10 highly produced species and provides commentary on barriers and future opportunities to achieving this goal.

## Introduction

Fish, shellfish and crustacean production via aquaculture has a critical role in meeting the future human demand for animal protein. The global demand for seafood has grown constantly over the past few decades, and in the current context of global population expansion and economic development, this rise in demand is expected to continue (Naylor et al., 2021a). Since wild-capture fishery production is widely considered to have plateaued, aquaculture has a rapidly increasing role in supporting global food security (Naylor et al., 2021b). Fish production via aquaculture is now approximately equal to capture fishery production for the first time in history, and extrapolation of the production trends highlights that aquaculture will be the dominant source of aquatic food during the coming decades (Naylor et al., 2021a). Global production of fish, crustaceans, molluscs and other aquatic animals reached 177.8 Mt in 2019. Of this total, capture fishery production was 92.5 Mt (a decrease of 4.3% from the previous year), while aquaculture production was 85.3 Mt (an increase of 3.7% from the previous year) (FAO, 2021).

While aquaculture as a whole has demonstrated consistent annual growth in production over recent decades, the species and systems underpinning this production are highly variable in technology level. A striking example is the major variability in the use of selective breeding and genetic improvement methods (hereafter referred to as ‘genetic technologies’). Application of genetic technologies to improve farmed animals and crops is considered a cornerstone of sustainable production (Georges et al., 2019, Li and Yan, 2020). Informed by the success of well-managed selective breeding programmes in terrestrial livestock, the first commercial-scale family-based selection programmes were developed for Atlantic salmon in Norway in the early 1970s (Gjedrem, 2010). The methods pioneered in salmon have since been applied in some form to several aquaculture species, albeit large-scale high-tech breeding programmes are restricted to the largest and most valuable sectors (e.g. salmonids, shrimp, tilapia, and certain marine finfish species) (Zenger et al., 2019, Houston et al., 2020). Such programmes have been demonstrably successful, for example resulting in genetic gains for growth rate of 13% per generation on average (Gjedrem and Rye, 2018).

Since the turn of the century, the breeding goals of advanced aquaculture programmes have broadened significantly and now include a significant focus on the improvement of host resistance to several infectious diseases (Boudry et al., 2021). However, this level of sophistication in genetic technologies is rare, and the vast majority of aquaculture production stems from species early in the domestication process, with typically very basic stock management (Teletchea, 2021). As a pioneer of this field, Professor Trygve Gjedrem commented in 2012 on the low uptake of selective breeding in aquaculture “To me this represents a tragic situation because the benefits from selective breeding are not reaching about 90% of the production. This loss affects farmed animals, producers and consumers.” (Gjedrem, 2012). While the uptake of genetic technologies in aquaculture has increased in the past 10 years, this major challenge still remains for the bulk of the global aquaculture industry. This review will focus on the status of genetic technologies in aquaculture, particularly the world’s top 10 most highly produced aquaculture species, summarising the state of the art together with opportunities and barriers to uptake. The review will then explore future technologies and directions, including discussing different routes to realising the benefits of genetic improvement.

## Summary of global aquaculture production

To discuss the impact and potential of genetic technologies in aquaculture, it is first important to gain perspective on the geography and species underpinning global production. Asia dominated the world's aquaculture production of fish and shellfish in 2019 (∼90% by volume), and the top ten aquaculture producers (excluding aquatic plants and non-food products) were China (48.2 Mt), India (7.8 Mt), Indonesia (6.0 Mt), Vietnam (4.4 Mt), Bangladesh (2.5 Mt), followed by Egypt (1.6 Mt), Norway (1.5 Mt), Chile (1.4 Mt), Myanmar (1.1 Mt), and Thailand (1 Mt). The majority of food production via aquaculture is finfish, comprising 56.3 Mt (66.0%). In comparison, there were 17.6 Mt of molluscs (20.6%) produced per annum, 10.5 Mt of crustaceans (12.3%) and 1.0 Mt of other aquatic animal species (1.0%). Most aquaculture production is from inland, freshwater systems, comprising 48.4 Mt (56.7%). Unlike terrestrial agriculture where relatively few major species dominate production (80% of global livestock meat production is derived from three species), a much broader and substantially more evolutionary diverse set of species underpin global aquaculture (80% of global aquaculture production is derived from approximately 70 species) (Houston et al., 2020). This diversity is highlighted within the top ten most highly produced aquaculture species globally which contains finfish, crustaceans, and molluscs (Table 1), and means that genetic technologies and their applications need to be tailored to species with substantial variation in methods of reproduction and other biological features.

**Table 1:** Overview of status of use of genetic technologies in production and research for the world’s top 10 aquaculture species by production volume.

Species (latin name, annual production volume in million tonnes)	Typical production systems	Major producing countries (top 5, Food and Agriculture Organisation Aquaculture production data, 2019)	Main sources of seed for production	Selective breeding programmes (public or commercial)	Status of genetic research (see also preprint by Nguyen, 2021)	Traits studied	Reference genome / genotyping tools	Level of selective breeding
Grass carp (*Ctenopharyngodon idella, 5.7)*	Semi-intensive and intensive culture in ponds, pens and cages in open waters	China	Hatcheries	-	Genetic parameters (2) (Huang et al., 2015, Peng et al., 2018) QTL analysis (2) (Huang et al., 2020a, Huang et al., 2020b, Yu et al., 2020)	Growth and morphometric traits, resistance to grass carp reovirus (GCRV)	Chromosome-level genome assembly (Wu et al., 2022)	1–2
Whiteleg shrimp (*Penaeus vannamei, 5.4)*	Intensive (Asia) and extensive (America) culture in ponds and recirculating systems	China, India, Indonesia, Ecuador, Vietnam	Hatcheries	Commercial selective breeding programmes focussed on growth and disease resistance	Genetic parameters (8) (Castillo-Juárez et al., 2007, Lu et al., 2016, Lu et al., 2017, Trang et al., 2019, Giang et al., 2019, Luan et al., 2020, Lillehammer et al., 2020) QTL analysis (5) (Yu et al., 2015, Huang et al., 2020a, Huang et al., 2020b, Peng et al., 2020, Zeng et al., 2020, Jones et al., 2020)	BW, resistance to White Spot Syndrome Virus, body colour, ammonia tolerance, pH tolerance, sex determination, nitrite tolerance	Genome assembly (scaffolds) (Zhang et al., 2019) 50 K SNP array, (Garcia et al., 2021)	3–4
Silver carp (*Hypophthalmichthys molitrix, 4.8)*	Mono and polyculture in ponds and cages	China (India, Bangladesh)	Hatcheries	Public: WorldFish Silver Carp Genetic Improvement Program (WSCGIP) (Hamilton et al., 2021)	Genetic parameters (1) (Gheyas et al., 2009) QTL analysis (2) (Wang et al., 2019, Zhou et al., 2020)	Harvest weight, harvest length, sex determination	Chromosome-level assembly (2022).	1–2
Nile tilapia *(Oreochromis niloticus, 4.6)*	Semi-intensive and intensive culture in ponds and cages.	China, Indonesia, Egypt, Brazil, Thailand	Hatcheries	Public and commercial advanced selective breeding programmes	Genetic parameters (14) (Rutten et al., 2005a, Rutten et al., 2005b, Charo-Karisa et al., 2005, Charo-Karisa et al., 2006, Lozano et al., 2013, Yoshida et al., 2017, Shoemaker et al., 2017, De Verdal et al., 2018, Workagegn et al., 2020, Mengistu et al., 2020, Mengistu et al., 2021, Barría et al., 2021a, Barría et al., 2021b, da Cardoso et al., 2021 QTL analysis (12) (Eshel et al., 2011, Eshel et al., 2012, Lühmann et al., 2012, Palaiokostas et al., 2013, Palaiokostas et al., 2015, Liu et al., 2014, Gu et al., 2018, Jiang et al., 2019, Cáceres et al., 2019, Zhu et al., 2021)	BW, fillet yield, surface area, Growth and morphometrix traits, resistance to tilapia lake virus, resistance to *Streptococcus iniae* and *agalactiae*, feed efficiency, cold tolerance, sex determination, swimming performance, female reproductive traits, male proportion, ammonia–nitrogen tolerance, temperature-dependant sex reversal, salinity tolerance	Chromosome-level assembly (Tao et al., 2021) 65 K SNP array (Peñaloza et al., 2020) 58 K SNP array (Joshi et al., 2018) 61 K SNP array + shrimp	2–4
Common carp *(Cyprinus carpio, 4.4)*	Semi-intensive or extensive mono and polyculture pond systems	China, Indonesia, Myanmar, Vietnam, Bangladesh	Hatcheries	Multiple synthetic breeds (especially in China) and some public sector family breeding programmes	QTL (12) (Chen et al., 2021)	Growth and morphometric traits, feed efficiency, eye shape, sex determination, scale pattern, polyunsaturated fatty acid content, intermuscular bone counts, body shape, carcass weight, resistance to cyprinid herpesvirus 3	High-throughput SNP array, 250 000 SNPs (Xu et al., 2014) Chromosome-level assembly (2021)	1–3
Manila clam (*Ruditapes philippinarum, 4.0)*	Intensive and extensive mono and polyculture systems	China	Hatcheries and harvested wild seed	Mass selection for growth (Liang et al., 2019)	Genetic parameters (5) (Zhao et al., 2012, Yan et al., 2014, Huo et al., 2017, Liang et al., 2019, Smits et al., 2020) QTL analysis (1) (Nie et al., 2017)	Growth and morphometric traits, parasite load (*Perkinsus olseni*), shell colour	Chromosome-level assembly (Yan et al., 2019)	1–3
Catla *(Catla catla, 3.3)*	Mono and polyculture in ponds and to some extent in oxbow lake culture systems	India	Hatcheries	Central Institute of Freshwater Aquaculture (CIFA) (Das Mahapatra et al., 2018, Sahoo et al., 2019) WorldFish (Hamilton et al., 2021) (Benzie et al., 2021)	-	-	Assembly level: scaffold (2020)	1–3
Bighead carp *(Hypophthalmichthys nobilis, 3.1)*	Extensive culture in open waters and pond-based polyculture	China, Iran (Islamic Rep. of), Lao People's Dem. Rep., Nepal, Myanmar	Hatcheries	-	QTL analysis (4) (Fu et al., 2016, Liu et al., 2016, Zhou et al., 2020, Zhou et al., 2021)	Growth and morphometric traits, sex determination	Chromosome-level assembly (2021)	1–2
Striped catfish (*Pangasianodon hypophthalmus, 2.7)*	Intensive mono and polyculture in ponds	Vietnam, India, Bangladesh	Hatcheries	National Breeding Centre for Southern Freshwater Aquaculture (NABRECSOFA) of Research Institute for Aquaculture No.2 (Vu et al., 2019a, Vu et al., 2019b) Research Institute for Fish Breeding (RIFB) Sukamandi, Indonesia (Tahapari et al., 2018)	Genetic parameters (5) (Sang et al., 2012, Tahapari et al., 2018, Vu et al., 2019a, Vu et al., 2019b, Dinh Pham et al., 2021)	Growth and morphometric traits, fillet weight, fillet yield, condition index, survival during the grow-out phase, resistance to *E. ictaluri* infection	Chromosome-level assembly (2019)	1–3
Atlantic salmon (*Salmo salar, 2.6)*	Intensive monoculture, marine and RAS	Norway, Chile, United Kingdom, Canada, Faroe islands	Hatcheries	Advanced commercial breeding programmes	Genetic parameters (>50), QTL analysis (>20)	Growth and morphometric traits, fillet quality, sexual maturation, resistance to several pathogens and parasites (see review (Fraslin et al., 2020)	Chromosome-level assembly (Lien et al., 2016), SNP arrays (Houston et al., 2014, Yáñez et al., 2016)	4

## Genetics and breeding in aquaculture

Widespread application of genetic technologies has major potential to help meet the continuously increasing global requirement for seafood. Selective breeding offers cumulative and permanent improvement in traits of economic, welfare and environmental importance. The proportion of aquaculture production derived from selective breeding programmes was estimated at 10% approximately a decade ago (Gjedrem et al., 2012). While this has increased in recent years, in particular in the global North, it is clear that selective breeding and genetic improvement of aquaculture species generally lag significantly behind the terrestrial animal and plant farming industries (Gjedrem et al., 2012, Houston et al., 2020). The majority of farmed aquatic species remain in the very early stages of a domestication process, and some seed is still sourced from the wild. Indeed, Teletchea and Fontaine proposed a five-stage process of domestication, ranging from 1 (First trials of acclimatisation to captive conditions) to 5 (Selective breeding programs are applied focusing on specific goals) (Teletchea and Fontaine 2014). The majority of aquaculture species are at level 4 (full life cycle is controlled in captivity without the use of wild inputs) or below the scale (88% of the 250 species included) (Teletchea, 2021). Therefore, it is clear that the missed opportunity for improvements in performance and reliability of production associated with well-managed selective breeding programmes still exists. Due to the recent domestication, it is logical that substantial standing genetic variation for production-relevant traits exists which, when combined with typically high fecundity of aquatic species, means major potential for rapid and sustainable genetic gains. Indeed, data gathered on the performance of aquaculture breeding programmes to date support this, for example for growth rate, an average of 14% gain per generation for finfish and 10% per generation for shellfish has been observed (Gjedrem and Rye, 2018). Encouragingly, comparable figures have also been observed for disease resistance in salmonid and shrimp species (11–19%) (Gjedrem and Rye, 2018).

While it is evident that fully closing the lifecycle in captivity is a very challenging milestone for many aquatic species, other features of aquaculture species biology are highly amenable to the application of genetic technologies in well-managed breeding programmes. For example, most species are highly fecund, meaning the potential for high selection intensity and therefore genetic gain with minimal inbreeding. For example, an Atlantic salmon female may produce circa 20 000 eggs, marine finfish such as sea bream hundreds of thousands of eggs per female, and shellfish such as oysters, millions of eggs per female. This fecundity is orders of magnitude higher than for terrestrial livestock, and this presents opportunities to design breeding programmes in different ways, with some similarities to crop species. External fertilisation is often possible which can give flexibility to the design of breeding programmes. For example, powerful tests of genotype by environment interaction can be achieved by splitting full-sibling families into groups and sending each group to a different environment for trait recording. The same so-called sib-testing method allows traits which are difficult or impossible to measure on the selection candidates themselves (e.g. disease resistance, fillet quality) to be recorded on very close relatives, facilitating accurate breeding value estimation. However, such reproductive features can also present significant challenges, for example in mass-spawning marine species, where uneven parental contribution and difficulty in tracking individuals during early life require tailoring the breeding programme design accordingly to avoid inbreeding.

As with the five levels of domestication proposed by Teletchea and Fontaine (2014), the use of genetic technologies can also be categorised according to their level of sophistication and potential to advance production as follows:

• **Level 1**: Seed for production is supplied from wild sources, or hatcheries with limited or no genetic management of stock.
• **Level 2**: Seed supplied from more advanced hatcheries which perform genetic management of stocks to promote genetic diversity and may include some basic directional selection (e.g. for growth of selection candidates).
• **Level 3**: Seed supplied from selective breeding programmes with routine recording of pedigree (by tagging and/or genotyping) to enable estimation of breeding values for production traits via family selection, with a focus on a small number of key traits in the breeding goal.
• **Level 4**: Seed supplied from more advanced selective breeding programmes with a focus on sibling testing, targeting many traits in the breeding goal via a selection index, and routine application of genomic tools, including for marker-assisted and/or genomic selection.

These levels will be referred to hereafter in the review, including the approximate placement of some of the world’s most highly produced species onto this scale (Table 1).

The factors that drive progression along this genetic technology scale in any given species or market are complex. For example, for a company, industry, or public sector organisation to embark on an organised and advanced breeding programmes, its leaders must (a) be convinced of the technical and performance advantages of the programme, (b) have the funds to invest in the venture over a substantial timeframe, and (c) identify and use the appropriate technical expertise for its development and execution. As a result, the process is typically gradual and can take many years (Chavanne et al., 2016). However, the benefits of prudent and continuous investment into genetic technologies are cumulative, permanent, and impact throughout the entire production chain.

## Genetic technologies in global aquaculture species

The extent of application of genetic technologies across the world’s top 10 produced aquaculture species is highly variable. As illustrated in Table 1, many highly produced species are still predominantly not genetically improved via selective breeding programmes, with producers typically relying on seed provided from basic hatcheries. In contrast, species such as Atlantic salmon, whiteleg shrimp and Nile tilapia have substantial production from advanced breeding programmes, with several generations of cumulative genetic improvement for growth and other target traits. There is a tendency for higher-value species to have more advanced status in genetic technologies. This review focussed on the world’s top 10 produced species by volume, but there is substantial overlap with the world’s top 10 species by value (FAO, 2021), so the same general conclusions and discussion apply.

### Atlantic salmon

In many technical aspects of aquaculture production, the Atlantic salmon industry is the most advanced globally. This includes use of the most advanced genetic technologies, with advanced (level 4) commercially viable breeding programmes supplying the bulk of the industry (see Table 1). The first commercial-scale salmon farming was in Norway in the 1960s, with trials of family-based breeding programmes following shortly after in the early 1970s (Gjedrem et al., 2012). These trials were based on Atlantic salmon genetic material originating from ∼ 40 Norwegian rivers, and were used in trials to obtain genetic parameters for important production traits, leading on to the first commercial breeding programme (Gjøen and Bentsen, 1997). Thereafter, a number of similar domestication events and breeding programmes were established, with strains such as the Mowi, the Rauma, the Jakta and the Bolaks originating from various sampling events and locations (Glover et al., 2017). The vast majority of global salmon aquaculture is still based on these original strains, including salmon farming in Chile and UK, following several crossing and international export events. There are also some North American-derived Atlantic salmon aquaculture strains which are predominantly farmed in the Australian (primarily Tasmanian) and Canadian aquaculture industries.

The continuous acquisition and consolidation of breeding companies has resulted in a small number of large international companies supplying seed to the majority of salmon-producing countries. These large breeding companies, which include Benchmark (UK), Aquagen (Norway, part of EW group) and Hendrix Genetics (Netherlands), tend to supply eggs to the market via separate country-specific breeding programmes, although there is also significant seed supply across countries (e.g. from Iceland to UK or North America). Mowi is one of the world’s largest Atlantic salmon producers and runs its own integrated level 4 breeding programme. These level 4 commercial breeding programmes focus on the simultaneous improvement of many traits, including resistance to several specific pathogens and parasites. In line with the advanced status of the industry, the research and development programmes linked to salmon genetic technologies are also substantial. This has enabled the development and routine application of genomic tools to augment the family selection, with an early success being marker-assisted selection linked to the discovery of a major quantitative trait locus (**QTL**) affecting resistance to Infectious Pancreatic Necrosis (**IPN**) (Norris, 2017). Subsequently, the development of high-density single nucleotide polymorphism (**SNP**) arrays (e.g. Houston et al., 2014, Yáñez et al., 2016) has enabled routine incorporation of genomic selection (D’Agaro et al., 2021), the primary benefit of which is the capture of the within-family (or Mendelian sampling) component of genetic variation in the typically large full-sibling families used. A large number of studies have now shown the benefit of using genomic prediction versus traditional pedigree prediction of breeding values, with an average increase in prediction accuracy across multiple aquaculture species of approximately 25% (Houston et al., 2020, discussed in more detail below).

The Atlantic salmon breeding industry is a global exemplar, and in recent years, there has been significant technology and skill transfer towards other major aquaculture species which are progressing through the genetic technology scale. For example, it is notable that several of the same companies or groups of companies running the salmon breeding programmes now also run commercial breeding programmes for tilapia [Benchmark, Genomar (part of EW group)], whiteleg shrimp (Benchmark, Hendrix), and rainbow trout [Hendrix, AquaGen]. In the case of Hendrix and EW group, there are additional cross-species interests across livestock and aquaculture, with the companies running breeding programmes for several terrestrial livestock and poultry species.

### Whiteleg shrimp

Whiteleg shrimp aquaculture has grown rapidly over the last 20 years, replacing the previously dominant shrimp species, the giant tiger prawn (*Penaeus monodon*), in part due to its increased resistance to infectious diseases. This species currently represents over 75% of all shrimp aquaculture production; most of which takes place in Asian countries, but there is also significant production in Latin America, with Ecuador being one of the top producing countries globally. Domestication and selective breeding programmes began in the 1980s in the USA and Latin America (Alday-Sanz et al., 2020). The application of genetic technologies to whiteleg shrimp production is more heterogeneous and less well-documented than for Atlantic salmon. However, it is clear that well-managed selective breeding programmes are responsible for ever-increasing proportions of production in all global regions, with technology levels of 3 or 4 underpinning the majority of production. This expansion of shrimp breeding programmes has in part been driven by the devastating impact of diseases and the prospect of disease-resistant strains, for example due to issues arising from white spot syndrome, Taura syndrome or early mortality syndrome (Castillo-Juárez et al., 2015). Indeed, producers are frequently faced with a race to rear shrimp to market size before the onset of pathogen outbreaks, and premature harvests or mortalities cause a significant financial burden.

The uptake of genetic technologies in shrimp production has been intricately linked to biosecurity and avoidance of infectious diseases, with a historical emphasis on the provision of specific pathogen-free (**SPF**) seed from hatcheries. There is significant international trade and shipment of SPF seed, including from North American hatcheries into Asia, albeit information on the proportion of production derived from selective breeding programmes is difficult to find. The use of SPF seed as a biosecurity tool has been at least partially effective, but as breeding programmes have developed, the term has resulted in some confusion amongst producers as to the relationship between pathogen-free status and disease resistance (Alday-Sanz et al., 2020). In part, this is connected to early Latin American shrimp breeding programmes, where mass selection was performed based on survivors of disease outbreaks, leading to the development of more pathogen-resistant strains. The SPF term refers to health status only, and disease resistance [or specific pathogen resistance (**SPR**)], specific pathogen tolerant (**SPT**)] is a genetic characteristic of the supplied seed (Alday-Sanz et al., 2020). The supply of SPF seed has established the platform for the implementation of modern selective breeding programmes, with major commercial operations such as Benchmark and Hendrix Genetics now supplying shrimp germplasm with disease resistance to multiple pathogens, as well as other performance traits. As with the salmon industry, there is a mix of specialist breeding companies and vertically integrated programmes, for example with Charoen Pokphand Foods (**CP**, Thailand) running an advanced integrated breeding programme. There is also an extensive genomic toolbox of relevance for whiteleg shrimp (Table 1). Historically, it has been shown that there is a negative genetic correlation between fast growth and resistance to WSSV, which has resulted in an undesirable trade-off for producers (Trang et al., 2019), an issue that may be effectively tackled via level 3 and level 4 breeding programmes. The use of family selection augmented by genomics will also lead to new opportunities to help address genotype by environment interaction which is a pressing issue when germplasm is distributed globally. This is particularly pertinent in shrimp breeding where there is substantial inter- and intra-country variation in key production parameters such as temperature, disease pressures, salinity, or stocking density. Depending on the specific circumstances of the breeding programmes and the producers they supply, this will be via product development of differentiated strains tailored for specific environments, or inclusion of general robustness / resilience traits into the breeding goal (Mulder, 2016).

### Carp and catfish species

Carp and catfish species represent a huge component of global aquaculture production, making up half of the top 10 most highly produced species globally (Table 1). These species are typically reared in extensive or semi-intensive freshwater systems, and particularly in Asia. Often several aquaculture species, and a mix of aquaculture and crop species, are reared together in polyculture pond systems. Carp species were some of the earliest to be at least partially domesticated and farmed for several thousand years. During the time since, there has been extensive historical hybridisation and development of breeds with specific characteristics (e.g. colour, scale pattern). However, these highly produced species remain amongst the least advanced in terms of the application of genetic technologies, and associated genetic tools and research, typically at Level 1 or Level 2 (Table 1). The common carp is arguably the most advanced of these species in terms of application of genetic technologies, extent of genetic research, and the genomic toolbox [reviewed in (Chen et al., 2021)]. European and Asian common carp strains have been farmed in both continents for over two thousand years, with the development of a wealth of strains or varieties (Chen et al., 2021). China has been especially active in breeding carp in recent decades and has developed multiple carp strains tailored for different environments and purposes (Hu et al., 2018) such as the Jian carp, a breed developed at the Chinese Academy of Fishery Sciences during the 1980s that currently represents over 50% of the Chinese production. The development of these strains has involved cross-breeding of ecotypes with different characteristics, gynogenesis, and some mass selection or family-based breeding programmes (albeit these tend to focus on a single trait (Hu et al., 2018)). For the other carp and catfish species in the global top 10 (silver carp, silver carp, catla, bighead carp, and stiped catfish), genetic technologies have not yet been applied to the same extent as for common carp. However, there have been a number of publicly funded selective breeding programmes, an expanding genomics toolbox, and a number of studies looking at genetic parameters for performance traits (Table 1). It should also be noted that some of the most advanced genetic research and family-based breeding programmes are in place for Rohu carp (*Labeo rohita*) (Rasal and Sundaray, 2020), which is particularly important in India, but does not make the top 10 global list. In general, there is little evidence for any of these carp species that commerciallydriven advanced breeding programmes focussed on multiple traits (level 4) are in progress. As such, it is likely to be these species where there is greatest potential for the benefit of application of genetics to enhance global aquaculture production and food security. However, implementing technicallyadvanced breeding programmes is expensive and time-consuming, and for species with relatively low economic value that are farmed in extensive or semi-intensive systems, it is unclear what or who will drive the financial and time investment required. As has been the case for several of the species, national or international public sector programmes are likely to play a role (Table 1), and investment into genetic technologies for such aquaculture species could be considered an excellent use of resources to address economic and food insecurity issues. However, breeding programmes are long-term ventures, and realisation of their benefits is relatively slow, and therefore, the sustainability of reliance on public sector investment is uncertain. The issues of funding and organisational models are discussed further in the section ‘Practical routes to genetic improvement’ below.

### Nile tilapia

Nile tilapia is one of the most important aquaculture species globally and is a critical source of quality protein and nutrients in several low- and middle-income countries. The success of tilapia aquaculture has been attributed in part to their ability to easily adapt to their farmed environment, displaying hardiness, rapid growth, and tolerance to a broad range of environmental conditions (Yáñez et al., 2020). The production systems of Nile tilapia are highly variable, with a broad range of (semi) intensive systems ranging from abundant small holder farms through to large-scale commercial production akin to advanced shrimp and salmon industries. While this variability in production systems is associated with substantial variation in the application of genetic technologies, selective breeding of tilapia is considered one of the highest profile success stories in aquaculture, via the development of the ‘Genetically Improved Farmed Tilapia’ (**GIFT**) strain. The GIFT strain exhibited an 86% increase in growth rate compared to the base population in just five generations (Bentsen et al., 2017). The GIFT strain broodstock were sampled from four populations of Nile tilapia reared in the Philippines (these originally being derived from Israel, Singapore, Taiwan and Thailand) as well as four wild populations imported from Africa [Egypt, Ghana, Kenya and Senegal (Gjedrem et al., 2012)]. The core GIFT breeding programme was run by WorldFish, but GIFT was widely distributed and now dominates world tilapia aquaculture production. This includes GIFT-derived strains used in major commercial breeding programmes (Spring Genetics based in Florida is part of Benchmark, and Genomar based in Norway is a company within the EW group). This market for genetically improved material from level 4 breeding programmes highlights the increasing maturity of the tilapia industry, particularly in certain South American and South-East Asian markets, with expanding markets in Africa. In addition, there are several large tilapia producers which undertake internal selective breeding programmes, including those managed by external genetic service companies such as Regal Springs based in Indonesia, Honduras and Mexico (“Regal Springs brings in Benchmark to advance tilapia breeding programme | The Fish Site”), and FirstWave group based in Africa (“Xelect and FirstWave in tilapia breeding partnership - Fish Farmer Magazine”). Such genetic management partnerships typically involve an arrangement based on fee-for-service and/or royalty based on sales. This model can provide an opportunity for producers to benefit from genetic improvement by gaining access to quantitative genetics and selective breeding expertise (discussed further in the section ‘Practical routes to genetic improvement’ below).

### Bivalve shellfish (including Manila clam)

Manila clam is the only bivalve which features in the global top 10 by production volume (Table 1) but bivalve aquaculture is a major component of seafood production in all regions of the world. Genetic technologies are generally underutilised in bivalve shellfish relative to major finfish and crustacean species, and this is the case for Manila clam. There have however been studies into the genetic basis of production traits, genomic tools developed, and moves towards the development of commercial breeding programmes (Smits et al., 2020). In part, this reflects the ease of obtaining abundant wild seed, and the very low individual value of each animal (Nascimento-Schulze et al., 2021). The reproductive biology of bivalves is quite distinct from most other aquaculture species, with broadcast spawning and exceptionally high fecundity being major features. The larvae also tend to experience extremely high mortality, which may in part be due to the high incidence of deleterious mutations in their genome and also causes major segregation distortion at nuclear loci (Plough, 2016). Bivalve species also tend to show lower than expected levels of inbreeding depression for any given mating structure (Plough, 2016). However, despite these challenges, there has been substantial effort and investment into bivalve selective breeding programmes, particularly in North America, Europe, and Australasia (Hollenbeck and Johnston, 2018, Potts et al., 2021). Despite the clear differences between the genetics and reproduction of bivalves and finfish, the majority of these breeding programmes have followed similar principles and used family selection. It may be that, as improved understanding of reproduction and inheritance increased in bivalves, the optimised design and use of genetic technologies may be quite distinct. For example, the ratio of within-family vs between-family variation is likely to be considerably higher in bivalves than in most vertebrates, suggesting that capturing this variation via genomic selection would provide relatively larger gains than in most species. It may also be the case that higher selection intensity is plausible without risking deleterious consequences of inbreeding. As with some of the carp and catfish species, the large-scale commercial investment into genetic technologies for bivalves has not yet been evident. Breeding programmes have been driven by public sector and academic initiatives, and some cooperative organisations of the farmers. However, with some specialised commercial breeding programmes emerging (e.g. Pacific Hybreed in the USA and SpatNZ in New Zealand), the signs are that genetic technologies are infiltrating bivalve aquaculture industries and will play an increasing role in sustainable seed supply and improved production traits in future years.

## Genetic technologies and disease control

While it is clear from the description of the major species above that growth rate tends to be the primary target trait initially for genetic improvement, breeding programmes operating at levels 3 and 4 can place significant focus on disease resistance. Infectious diseases continue to be one of the most pressing issues affecting the sustainability of the global aquaculture industry, particularly for intensively farmed species (Pérez-Sánchez et al., 2018). The role of genetic technologies in combatting infectious diseases in aquaculture is particularly important compared to terrestrial systems because (i) prevention of exposure to pathogens using biosecurity measures is challenging in most production settings due to constant exposure to the open water environment, (ii) vaccination and treatment measures are often not readily available and tend to be logistically challenging to implement, and (iii) the aforementioned high fecundity of aquaculture species means that strains with high genetic resistance can be disseminated to production systems rapidly to have a major impact (Houston, 2017).

The sib-testing systems possible in level 3 and level 4 breeding programmes are also highly amenable to performance testing of close relatives of selection candidates (which are usually retained in a largely biosecure breeding nucleus), and this testing routinely includes disease challenge models in the more advanced industries (e.g. salmon, shrimp, tilapia). The flagship example of the control of Infectious Pancreatic Necrosis in Atlantic salmon using marker-assisted selection has highlighted the potential of genetic technologies as a solution to disease (Norris, 2017). It is critical therefore that the fields of aquaculture genetics and health are considered in tandem; sustainable control of problematic infectious diseases requires investment and uptake into genetic technologies, and species or industries without level 3 or level 4 breeding programmes do not have this weapon in their arsenal. However, it is also a two-way relationship as highlighted with the SPF shrimp described above, industries relying on extensive shipment of genetically improved germplasm need stringent biosecurity to avoid spreading pathogens. Furthermore, each proposed means of tackling infectious diseases should not be considered in isolation, and use of genetic technologies should form part of an integrated disease control strategy that includes attention to other prevention and treatment measures (Barrett et al. 2020). Indeed, the interaction between genetic resistance and vaccination is a promising area to study in order to optimise the additive effects of both approaches.

### What can genomics offer?

In the 21st century, genomic tools have developed rapidly, and certain aquaculture industries have been near the forefront of their application to farmed animal production. The rapid adoption of molecular genetic markers into aquaculture breeding is partly because the use of genotyping for family assignment and pedigree recording has been commonplace for some industries for many years (Vandeputte and Haffray, 2014). For some species (e.g. mass-spawning marine species), separation of families at spawning is not possible, and therefore, genotyping for parentage assignment is a requirement. Also, even if family separation and rearing is possible, it requires significant infrastructure and time investment, and results in some common environment effects. Therefore, genotyping for parentage in mixed-family systems can be a more effective approach, especially if genomic information is used downstream in breeding decisions via genomic or marker-assisted selection.

A second reason for the relatively rapid interest and uptake of genomics technologies into aquaculture breeding has been the successful identification and use of markers linked to the major IPN resistance QTL described above. This use of marker-assisted selection helped the global salmon industry buy-in to the value of molecular genetics, and the role of genetics in general as part of the solution to health challenges (Moen et al., 2015, Norris, 2017, Houston et al., 2020, Pavelin et al., 2021). However, while major QTL have also been discovered for certain other production traits, including resistance to other pathogens (reviewed in Fraslin et al., 2020), it is clear that the IPN example is unusual due to the large proportion of the genetic variation explained by the QTL. Therefore, as routine high-density genotyping technologies developed over more recent years – for example SNP arrays and genotyping by sequencing (Robledo et al., 2018) – the focus of using genomic information in selection decisions has shifted somewhat. Following on from its transformation of selective breeding in some terrestrial livestock species, genomic selection has become commonplace in aquaculture breeding (Zenger et al., 2019, Houston et al., 2020) and has been demonstrated empirically to result in improved realised genetic gains (e.g. Lillehammer et al. 2020). The primary benefit in the typical sib-testing schemes of aquaculture programmes is the capture of the within-family (aka Mendelian sampling) component of genetic variation. This is particularly valuable for aquaculture species compared to terrestrial livestock, because of their high fecundity and large full-sibling family sizes. Therefore, most level 4 breeding programmes, including all major salmon breeding programmes, are using genomic selection. The benefits to selection accuracy in a typical aquaculture species scenario versus family selection alone are on average 20–25% for both growth and disease resistance (Houston et al., 2020). There are other advantages relating to improved control of inbreeding, and potentially,a reduction in generation interval (albeit the latter is mostly applicable to progeny testing which is not routinely performed in aquaculture).

To date, the use of genomics has enabled the establishment of level 3 and level 4 breeding programmes in species where physical family tracking is difficult or impossible (e.g. European sea bass, gilthead seabream), and also for improved rates of genetic gain in the most advanced breeding programmes (e.g. those for salmon, shrimp, tilapia). The workhorse genotyping tool for genomic selection is a SNP array, and these are only available for four of the top 10 most produced aquaculture species (Table 1). A related hindrance to realising the benefit of genomics for genetic improvement of other species is the cost – collection of individual genome-wide SNP data on large populations of animals can be prohibitively expensive. Furthermore, evidence to date suggests that the benefits of genomic prediction are only observed when the reference populations are closely related to the validation population (or selection candidates) (Palaiokostas et al., 2019, Fraslin et al., 2022). Therefore, annual performance testing is typically required, and the associated costs for large numbers of animals is very high.

Solutions to reduce the cost of genomic tools to aquaculture breeding programmes are important to facilitate broader uptake globally, and to democratise genomics and its benefits. Several potentially complementary strategies have been explored to this end. Firstly, since per-unit cost of a SNP array is dependent on the order volume, combining SNPs from multiple species onto a single array is cheaper, for example, the recentlydeveloped combined European sea bass and sea bream array (Peñaloza et al., 2021), and the equivalent for serrasalmid fish (Mastrochirico-Filho et al., 2021). This approach can be supported by collaboration between companies, and/or harnessing the order volume of the most advanced species (e.g. salmon) to support more affordable prices for emerging species. Secondly, it is possible to design lower-density SNP panels and genotyping platforms that typically have a lower per-unit cost; a wealth of studies have shown that reducing SNP density down to approximately 5,000 SNPs or lower still results in near maximum prediction accuracy (Zenger et al., 2019, Kriaridou et al., 2020, Houston et al., 2020). A third promising avenue is the use of genotype imputation, which infers untyped SNPs in populations genotyped at lowdensities from a reference population genotyped at a higher density. This strategy allows for the majority of individuals to be genotyped with a low-density SNP panel, potentially with only a few hundred SNPs. Recent studies have suggested that this approach may be feasible with minor, or no, loss of prediction accuracy (Tsai et al., 2017, Yoshida et al., 2018, Tsairidou et al., 2020).

These low-cost genotyping approaches can facilitate the use of combined-purpose SNP panels to support developing aquaculture breeding programmes. For example, the few hundred SNPs used as the basis for genotype imputation could also be applied for pedigree construction in mixed-family environments. This in turn may save on labour and infrastructure costs associated with tagging and/or separate rearing of families. Those same SNP panels could also be enriched for known QTL-linked markers for major effect QTL identified in the species of interest, meaning that marker-assisted selection could be combined with (genomic) breeding value estimation to further improve genetic gain for target traits. In this sense, multi-purpose SNP panels may be an enabling tool to support design and establishment of level 3 and level 4 breeding programmes from the outset, rather than a sequential add-on to an existing programme. As such, genomics has a significant role to play in supporting the uptake of genetic technologies in aquaculture, and continued efforts towards low-cost and practical genotyping approaches are essential.

## Practical routes to genetic improvement

The factors influencing the uptake of genetic technologies for a particular species or geography are complex, depending on factors such as the value and market for the product, whether farming is extensive or intensive, and the economic and socio-cultural situation. Private sector commercial breeding programmes are currently limited to relatively few species, albeit this number is growing as the importance of genetics in production is increasingly recognised. The sheer number and the diversity of species comprising global aquaculture present challenges, due to the requirement to develop programmes and tools tailored to the biology of each species (Houston et al., 2020). Commercial organisations involved in selective breeding in aquaculture tend to fall into one of three categories. The first are specialised breeding companies whose business is to sell genetically improved eggs to producers, and these are most evident in Atlantic salmon (e.g. Benchmark, Aquagen, Hendrix) and are typically linked to equivalent companies for other sectors including tilapia and shrimp. Such specialised breeding companies do not yet exist yet for most other aquaculture species, likely because the added value of genetically improved germplasm is not yet sufficient to support a profitable business. There are also some fully integrated companies where their breeding programme supplies only their own producers, examples of which are Mowi in salmon, and CP for shrimp. Finally, there are also specialist genetic services companies which have a major role across a broader sphere of aquaculture species. These companies manage breeding programmes on behalf of (typically) small- and medium-sized producers. Examples include Xelect (UK), Benchmark (formerly Akvaforsk Genetics Centre, Norway), and Center for Aquaculture Technologies (North America). These genetic service companies also typically perform molecular genetics (e.g. genotyping) and contract research services to support external clients.

In addition to the private sector, there are various academic, national, and international breeding programmes developed and supported with public funds. For example, WorldFish is an international CGIAR organisation who produce and disseminate genetically improved tilapia and various carp species, typically to low- and middle-income countries. As discussed above in the context of carp and catfish species, public sector investment into genetic technologies for aquaculture is likely to be a sound investment in the sustainability of food production for a given country or region (and play a significant role in moving species up the technology scale). However, noting that modern selective breeding programmes are continuous processes, resulting in cumulative and permanent improvements in production traits, the development of a specific strain with particular characteristics should not be considered an end point, as improvements in production traits can continue each generation with level 3 and 4 programmes. As such, the ongoing and stable commitment of resources are required to support these breeding programmes. Public-private partnerships may form part of the solution to achieving this sustainable investment into genetic technologies, leading to an increase in the proportion of the global aquaculture industry using level 3 or level 4 programmes.

## Future disruptive technologies

The past decade has seen significant focus placed on the use of genomics in aquaculture breeding, with some highly impressive results. As discussed above, genomics still has a major enabling role to play across many species and sectors and can help support fledgling breeding programmes from the outset. However, arguably any future innovation in the use of genomics to predict breeding values is likely to be leading towards improved efficiency of the process, for example lower cost genotyping and phenotyping. There may be incremental gains arising from use of functional genomics and prioritisation of putative functional variants in prediction models, but results from livestock breeding have been underwhelming to date. As such, the use of genomic and marker-assisted selection can be considered relatively mature technologies in advanced aquaculture breeding programmes. In the coming years, it is likely that particular emphasis will be placed on improving phenotyping technologies to provide more refined target traits for breeding goals (Fu and Yuna, 2022). For example, advances in imaging and sensor technologies can enable individual-level recording in production settings, which combined with artificial intelligence developments, could provide extensive - and potentially realtime - data to feed into breeding nuclei. Automatic imaging, machine vision, and image processing software have already been developed to record traits such as growth, sex, and meat quality, with substantial potential for health, feed intake, and behaviour traits (Fu and Yuna, 2022). Such data are likely to be critical to effectively tackling genotype by environment interactions, providing the opportunity for central breeding nuclei to either focus on tailored products for specific production systems or robust-performing strains across multiple environments. The routine large-scale disease challenge experiments discussed above, focussed on crude phenotypes such as mortality, are unlikely to be sustainable for the industry, in part due to increasing animal welfare concerns. Therefore, intermediate phenotypes and biomarkers, potentially from non– or minimally invasive measurements on selection candidates themselves, may provide new disease resistance indicator traits (Clark et al, 2020). Similar methods have the potential for precision management of aquaculture farm systems, for example by integrating genomic, epigenetic, biomarker, and environmental data to inform farm management decisions during the animals’ lifetime (Clark et al, 2020).

Genome-editing tools such as CRISPR-Cas have significant potential for applications to improve welfare and production traits in aquaculture and are likely to be widely adopted into the sector in the coming decade. In Japan, the sale of genome-edited tiger puffer and red sea bream – both of which have targeted mutations to improve growth rate – has recently been approved (Japan embraces CRISPR-edited fish, 2022). While early examples have focussed on growth, it is likely that traits related to animal welfare and reduced environmental impact are likely to gain significant traction in future. For example, there are major projects underway with the ultimate goal of using genome editing to transfer the innate mechanisms of resistance to sea lice from certain Pacific salmon species (e.g. coho) to the widely-farmed Atlantic salmon (e.g. CrispResist; https://nofima.com/projects/crispresist/). If successful, and a lice-resistant salmon strain is developed, then this solves a major industry barrier causing vast economic, welfare, and environmental problems.

Assuming the public and regulatory environment does permit genome editing to be applied in aquaculture production, there are two major technical hurdles to overcome, specifically the identification of the target edits and integrating editing technology into selective breeding programmes. There are a wealth of molecular and cellular technologies which can be used to identify potential targets, depending on the conceptional approach taken. Firstly, genome editing can be applied to modify target genes or mutations in known disease resistance QTL regions defined from GWAS approaches, potentially also using transcriptomic comparisons of animals carrying alternate alleles at the QTL. Where useful cell line models of host-pathogen interaction exist, it is possible to use CRISPR to perturb specific candidate genes and perform in vitro disease challenges [an approach taken for the IPN QTL (Pavelin et al., 2021)]. However, targets need not be limited to existing QTL, and genome editing can create *de novo* variation that can have a major impact via modification of a target gene’s activity in the absence of any known segregating QTL. This can be achieved using a hypothesis-driven approach; for example as was applied to modify a domain of the putative cellular receptor to develop complete resistance to the porcine reproductive and respiratory virus in pigs (Tait-Burkard et al., 2018). Alternatively, genome-wide pooled CRISPR screens such as the genome-wide knockout (**GeCKO**) approach can be used to identify *de novo* targets essential for host-pathogen interaction (Gratacap et al., 2019). Such approaches (used alone or in combination) can be applied to identify editing targets for downstream testing *in vivo*, where an assessment of edited animals versus their wild-type counterparts will inform on the impact on the target trait, and also any potential pleiotropic effects.

However, even when target edits are identified that have favourable impact on traits of interest, integrating this technology into aquaculture selective breeding programmes requires careful thought and technical development. Target edits will need to be introduced precisely, repeatably, and with high efficiency. Furthermore, it is essential that the process of introducing edits does not cause bottlenecks in the breeding programme, as genome editing is simply a potential tool in the toolbox of such programmes, not a replacement. The most widely-applied *in vivo* genome-editing method in aquatic species is microinjection into the early-stage embryo, and this typically results in a highly mosaic F0 animal (Gratacap et al., 2019). Multiple generations of breeding may be required to achieve fully homozygous animals, and this presents a significant barrier particularly for species with long generation intervals, such as Atlantic salmon. Furthermore, due to substantial variation in regulatory environments and customer preferences, introducing edits into the core breeding nucleus may be a risky approach, and editing in disemination or multiplication lines may be preferable. A promising avenue to help address some of these issues is the use of germ cell isolation and editing which, when combined with sterilised surrogate broodstock, could facilitate efficient and complete editing in the next generation (Jin et al., 2021). This germ cell and surrogate technology could also offer a route to decoupling editing from the breeding nucleus, if siblings of selection candidates are used as the source of the germ cells, and surrogates are viewed as multipliers. A further potential advantage of this approach is that edits causing sterility of the offspring [e.g. (Wargelius et al., 2016)] could be concurrently applied as a means of preventing any potential escapee fish interbreeding with wild conspecifics. As such technologies develop, it will also be important to consider how introducing new genetic variation via genome editing alters the genetic architecture of traits of interest, via epistatic interactions. As a hypothetical example, growth is widely considered as a polygenic trait in aquaculture populations, but the introduction of an edit to knockout myostatin (for example) is akin to introducing a major QTL. In this situation, selection for the target trait, or correlated traits, will need to account for epistasis – i.e. family ranking for the trait may be substantially different in lines carrying the edit versus those without. A side benefit of the use of gene editing to produce lines with favourable traits (e.g. complete resistance to a particular disease) is that less focus may need to be placed on that trait in the breeding goal. This means that increased selection intensity could be placed on other traits and would reduce the need for routine disease challenge experiments as part of the selection programme.

## Conclusion

The application of genetic technologies towards improving sustainable aquaculture production has increased rapidly in the early part of the 21st century. However, the technology level of seed supply remains highly variable across species, including the top 10 species by production volume globally. The Atlantic salmon industry has led the way, with advanced family-level programmes incorporating genomic selection for multiple traits. Assisted by company consolidation, similar major programmes are now in place for marine shrimp and Nile tilapia. However, seed supply of many of the highly produced freshwater aquaculture species (e.g. carps, catfish) is still based on fairly basic genetic technologies. Broader uptake of genetics in less valuable sectors may be assisted via integrated or cooperative breeding programmes for producers, public–private partnership, or with external genetic service management. Genomics can be an enabling tool to support genetic improvement, especially with multi-purpose marker panels to allow pedigree construction in tandem with low-cost marker-assisted and genomic selection. In the advanced aquaculture sectors, genomic selection is now nearing maturity and will become more efficient and lower cost over the coming years. This timeframe is also likely to see an increased focus on improving phenotypes, and associated high-throughput phenotyping and data capture technologies. Genome editing may be the next disruptive technology for the second quarter of the century, offering potential solutions to some of the industry’s biggest sustainability challenges. However, major effort is required to integrate genome editing into well-managed breeding programmes. Genetic technologies remain hugely underexploited, and addressing this should remain a priority to realise the potential contribution of aquaculture to global food security challenges.

## Ethics approval

Not applicable.

## Data and model availability statement

Not applicable.

## Author ORCIDs

**RDH:** 0000-0003-1805-0762

**CK:** 0000-0003-2277-2311

**DR:** 0000-0002-9616-5912.

## Author contributions

**RDH:** Conceptualisation, Writing – Original Draft, Writing – Review and Editing

**CK:** Conceptualisation, Writing – Original Draft, Writing – Review and Editing

**DR:** Conceptualisation, Writing – Original Draft, Writing – Review and Editing.

## Declaration of interest

None.

## Acknowledgements

None.

## Financial support statement

The authors were supported by Biotechnology and Biological Sciences funding to The Roslin Institute (BBS/E/D/20002172, BBS/E/D/30002275, and BBS/E/D/10002070).
